# Case report: Lamin A/C gene mutation in patient with drug-induced type 1 Brugada syndrome at high arrhythmic risk

**DOI:** 10.3389/fcvm.2022.1099508

**Published:** 2023-01-10

**Authors:** Vincenzo Russo, Giovanni Papaccioli, Valeria Maddaloni, Adriano Caputo, Nicola Pepe, Anna Rago, Michele Maiorino, Paolo Golino, Gerardo Nigro

**Affiliations:** ^1^Cardiology Unit, Department of Medical Translational Sciences, Monaldi Hospital, University of Campania Luigi Vanvitelli, Naples, Italy; ^2^Clinical Biochemistry Unit, Genetic Section, Monaldi Hospital, Naples, Italy; ^3^EP Department, Boston Scientific, Milan, Italy

**Keywords:** arrhythmias, Brugada syndrome, Lamin A/C mutation, flecainide challenge test, electroanatomical mapping, programmed ventricular stimulation, induced type 1 Brugada pattern

## Abstract

We report the case of drug-induced type 1 Brugada syndrome at high arrhythmic risk associated with Lamin A/C gene mutation.

## Introduction

The sudden cardiac death (SCD) risk stratification in drug-induced type 1 Brugada syndrome is still challenging. Current guidelines do not support the role of programmed ventricular stimulation (PVS) in this subset of patients. The genetic testing for SCN5A gene is the only recommended for probands with BrS (1); however, many other genes modulating the arrhythmic risk have been described in patients clinically affected by BrS (2). We report the case of drug-induced type 1 Brugada syndrome at high arrhythmic risk associated with Lamin A/C gene mutation.

## Case presentation

We report the case of asymptomatic 42-year-old male with evidence of 1 mm J-point elevation in V2–V3 and a saddleback shaped ST elevation V3 on basal electrocardiogram (ECG). His family history was positive for sudden cardiac death (father died at 64 years old) and dilatative cardiomyopathy (sister, onset at 40 years old). The patient had no personal relevant medical history.

**Figure 1:**
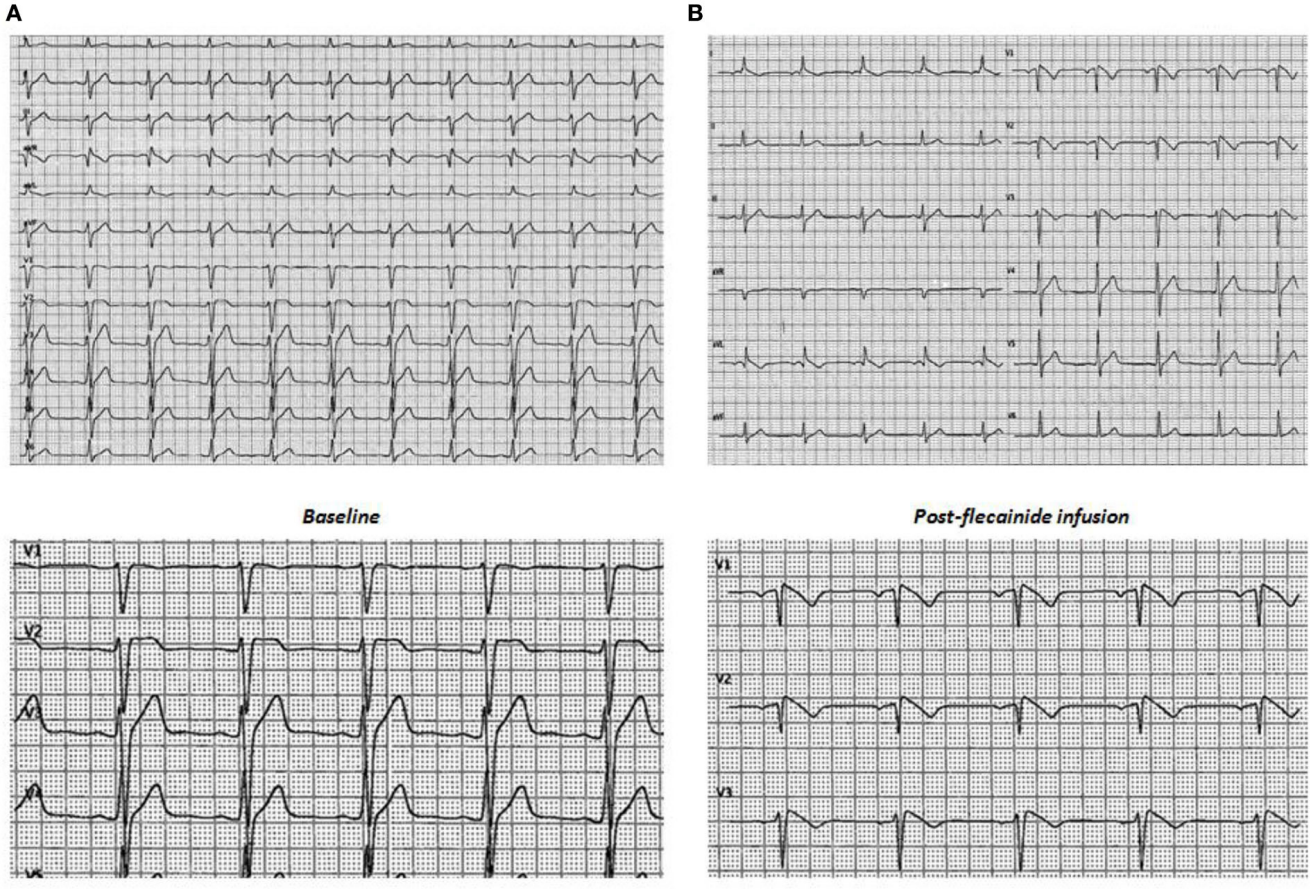
Basal **(A)** and post-flecainide infusion **(B)** twelve-lead electrocardiographic recordings.

Transthoracic echocardiography (TTE) showed no cardiac abnormalities. The sodium channel blocker test using flecainide 2 mg/kg over 10 minutes revealed a diagnostic type I Brugada ECG (Figure 1). For SCD risk stratification, an endocardial three-dimensional (3D) map of the right ventricle was constructed using a high-resolution mapping system (RhythmiaHdx™ Mapping System, Boston Scientific Corporation) and the programmed ventricular stimulation (PVS) at two ventricular sites, right ventricular apex (RVA) and right ventricular outflow tract (RVOT), with up to three premature extrastimuli was performed.

**Figure 2:**
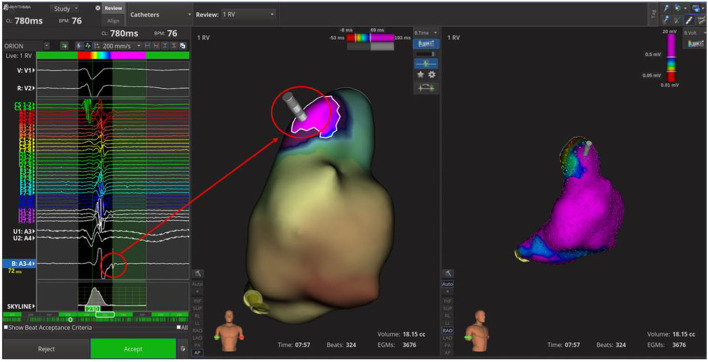
Time-domain electroanatomical map. In the antero-superior region of right ventricle outflow tract; the systolic late potentials were shown.

At unipolar (cut-off: 5.5–8.3 mV) and bipolar (cut-off: 0.2–1 mV; 0.5–1.5 mV) voltage mapping in sinus rhythm no abnormalities were shown; the propagation map instead showed systolic late potentials in the antero-superior region of RVOT, where depolarization slowed significantly (Figure 2; Supplementary Video 1).

**Figure 3:**
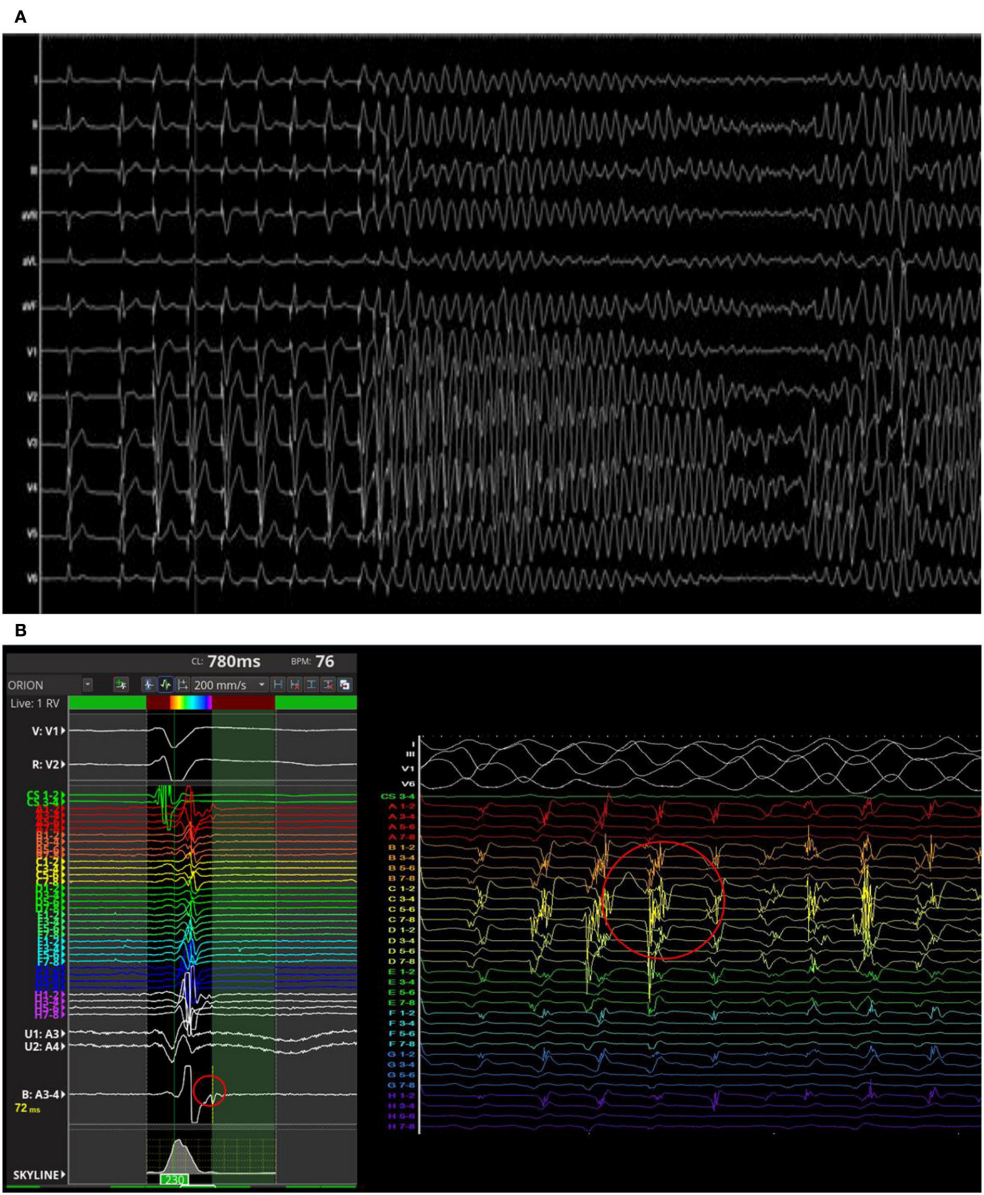
Polymorphic ventricular tachycardia induced by programmed ventricular stimulation from right ventricular outflow tract (RVOT) **(A)**. The early diastolic potentials were recorded by the mapping catheter placed in RVOT **(B)**.

The PVS from RVOT with a paced drive train at cycle length of 500 ms followed by two extrastimulus at 220 and 200 ms respectively revealed the induction of a sustained polymorphic ventricular tachycardia (PVT) symptomatic for syncope and treated by external DC-shock (Figure 3A). During the arrhythmic event, the early diastolic potentials were recorded by the high-density diagnostic catheter (IntellaMap Orion™, Boston Scientific Corporation, US) placed in RVOT (Figure 3B). Subcutaneous ICD implantation was performed in order to prevent the sudden cardiac death. At 6 months follow-up, the patient did not experience arrhythmic events. The molecular genetic analysis showed a c.1718C>T heterozygous variation on exon 11 of Lamin A (p. Ser573Leu). Family members were genetically screened and the probands' sister and daughter showed the same LMNA mutation in absence of ECG abnormalities.

## Discussion

We described the LMNA Ser573Leu missense mutation in asymptomatic drug-induced type 1 Brugada patient at increased arrhythimic risk for family history of sudden death and polymorphic VT induction at PVS. This missense mutation causes the substitution of a hydrophilic aminoacid (Serin) to a hydrophobic one (Leucine) in the highly conserved globular carboxyl tail of the Lamin A isoform, involved in the lateral assembly of protofilaments and mediating the Lamin network formation and may lead to change in the protein secondary structure, given the difference in polarity, electrical charge and dimension of the two peptides (3).

The LMNA Ser573Leu missense mutation was found in heterozygous individuals affected by dilatative cardiomyopathy, hypertrophic cardiomyopathy and atrio-ventricular block, familiar partial subtype 2 lipodystrophy, limb girdle muscular dystrophy, Charcot-Marie-Tooth disease, Emery-Dreifuss muscular dystrophy (3, 4).

Our case report firstly described its association with BrS and supports the hypothesis of a possible alteration of sodium ionic currents (INa) in cells carrying LMNA gene mutations. Salvarani et al. demonstrated a direct interaction between Lamin A/C protein and SCN5A gene promoter with a significative *in vitro* reduction in SCN5A expression in induced pluripotent stem cells K219T LMNA-mutated derived cardiomyocytes (5). Recenlty, Armaroli et al. described a case of 31-year-old man, carrying a heterozygous mutation in exon 4 of Lamin A/C (p.R216C), with spontaneous type 1 Brugada ECG pattern who experienced at-rest cardiac arrest (6). Based on this evidence, we suggest to perform the genetic testing for LMNA gene in all probands with BrS.

The present case offers us the opportunity to discuss the role of PVS in drug-induced type 1 Brugada syndrome. The 2022 ESC guidelines for the management of patients with ventricular arrhythmias and the prevention of sudden cardiac death (1) stated that PVS may be considered in asymptomatic patients with a spontaneous type I BrS; however, no indication was given for those with drug-induced type 1 BrS. The relatively low arrhythmic risk of this subgroup, it does not mean zero risk. In the IBRYD study including 226 drug-induced type 1 BrS patients, 4.9% of them experienced a primary outcome event (appropriate ICD therapy or SCD) during a median follow-up of 106 months (7). In a recent meta-analysis including 4.099 patients with a mean follow-up of 4.5 years, the pooled annual incidence of major arrhythmic events (MAE) was 0.65% in symptomatic and 0.21% in asymptomatic drug induced type 1 BrS patients; the incidence of MAE between symptomatic drug-induced and asymptomatic spontaneous Type 1 was similar (8). The PVS failed to stratify the high-risk drug-induced BrS patients, showing a low positive predictive value (8.9% in asymptomatic; 9.6% in symptomatic); however. it may be considered a good tool to identify those at low arrhythmic risk, showing a high negative predictive value (95% in asymptomatic; 100% in symptomatic). Recently, the electroanatomical mapping has been considered an additional tool for SCD risk stratification among BrS patients (9); in particular an extensive RVOT electroanatomical abnormalities identify asymptomatic BrS patients at high risk (10). In order to better characterize the BrS electrophysiological substrate, we usually propose the ventricular endocardial electroanatomical mapping for all BrS patients followed at our center.

Since the drug-induced type 1 BrS might be part of laminopathies spectrum, a patients' centered approach including LMNA genetic testing, high density electro-anatomic mapping and PVS should be applied for the sudden cardiac death risk stratification.

## Conclusion

Drug-induced type 1 BrS might be part of the laminopathies spectrum. The LMNA gene screening, the high density electro-anatomic mapping and the programmed ventricular stimulation should be considered in the patients' centered care of drug-induced type 1 BrS.

## Data availability statement

The original contributions presented in the study are included in the article/Supplementary material, further inquiries can be directed to the corresponding author.

## Ethics statement

The studies involving human participants were reviewed and approved by University of Campania Luigi Vanvitelli ID: 07082021. The patients/participants provided their written informed consent to participate in this study.

## Author contributions

VR: study concept and writing of the manuscript. PG and AR: critical revision of the manuscript for intellectual content. MM, VM, GP, AC, VR, and NP: acquisition of data and figures. All authors cared for the patient and contributed to the writing of the report. All authors contributed to the article and approved the submitted version.

## Supplementary material

Supplementary Video 1 Activation map during sinus rhythm. The antero-superior region of right ventricle outflow tract shows a delayed activation.
